# Epidemiological Profile of Non‐Hodgkin's Lymphomas Seen at Kinshasa University Clinics From 2012 to 2022

**DOI:** 10.1002/cam4.71254

**Published:** 2025-09-20

**Authors:** Mbwamulungu Nakweti Julia, Azako David, Pezo Serge, Bokambadja Fabrice, Kapour Kieng Germain, Bompangue Nkoko Didier, Kisile Olive, Lebwaze Massamba Bienvenu, Kabongo Mpolesha Jean‐Marie

**Affiliations:** ^1^ Department of Anatomy and Pathological Cytology University Clinics of Kinshasa Kinshasa Democratic Republic of the Congo; ^2^ One Health Institute for Africa University of Kinshasa Kinshasa Democratic Republic of the Congo; ^3^ Faculty of Medicine University of Kikwit Kikwit Democratic Republic of the Congo; ^4^ The National Cancer Center Democratic Republic of the Congo

**Keywords:** epidemiology, Kinshasa, non‐Hodgkin lymphoma

## Abstract

**Introduction:**

Non‐Hodgkin lymphoma (NHL) is a heterogeneous group of cancers whose global incidence has been increasing in recent years. In the DRC, the absence of a national cancer registry is a serious handicap to the epidemiological evaluation of NHL. There is still a lack of knowledge about this pathology among the population and health professionals. An observational survey of the country's hospitals is enough to note the absence of protocols for the management of NHL. The objective of this study is to determine the epidemiological aspects of non‐Hodgkin lymphoma in the DRC.

**Materials and Methods:**

A descriptive cross‐sectional study, conducted at the University Clinics of Kinshasa, from 2012 to 2022. The chi‐square correlation test is used to compare proportions with the 95% confidence interval (*α* = 0.05).

**Results:**

Out of a total of 2070 cancer cases, 51 NHL cases were recorded, or 2.5%. 98% of the recorded cases were B‐cell lymphomas compared to 2% T‐cell lymphomas. 80% of cases were aggressive and 20% were indolent. 55% of cases were male versus 45% female, with a sex ratio of 1.2. The age of the patients ranged from 3 to 80 years, with a mean age of 42 years and a median of 45 years. The histological type DLBCL was predominant in 63% of cases. 59% of cases involved lymph nodes.

**Conclusion:**

Non‐Hodgkin lymphoma (NHL) is a heterogeneous group of cancers whose global incidence has been increasing in recent years, with the outbreak of environmental and infectious factors. Knowledge of epidemiological aspects contributes to the improvement of the fight against NHL in the DRC.

## 1 Introduction

Non‐Hodgkin lymphoma (NHL) is a heterogeneous group of cancers whose global incidence has been increasing in recent years with the outbreak of environmental and infectious factors (HIV, EBV, HTLV‐I infection, and even certain bacteria such as *Helicobacter pylori* , radiation) that act on genetic factors [1, 2, 3, 4, 5]. It is estimated that the number of new NHL cases per year worldwide is expected to double from 2012 to 2030, or 15 new cases per 100,000 population [2, 6, 7].

Although the different forms of NHL have some things in common, in particular their lymphatic origin, they differ in their morphological, immunological, and molecular characteristics, their mode of development, and their impact on the body [8].

For patients, this translates into different symptoms, course, and response to treatments depending on the form of NHL they have [9].

NHL is the most common hematologic malignancy in the world, accounting for nearly 3% of cancer diagnoses and deaths. It accounts for about 80% of all lymphomas [1].

By frequency, NHL ranks 7th among cancers (9th place for cancer deaths) in the West [10, 11, 12].

The most common subtypes of non‐Hodgkin lymphomas are diffuse large B‐cell lymphomas (25%–30%) and follicular B‐cell lymphomas. B‐cell lymphomas account for 85% of NHL [2, 13, 14].

In terms of immuno‐phenotypic, the distribution varies according to geographical areas; in Europe, 80% of lymphomas are type B and 20% type T. According to the latest World Health Organization (WHO) classification, DLBCL is the most common type of NHL in Western countries, accounting for about 31% of adult cases. T‐cell lymphoma shows a higher incidence in African Americans [3, 4].

East Asian countries, especially Japan, as well as countries in West Africa and the Caribbean, have higher rates of human T‐cell lymphoma virus (HTLV‐1), and consequently a higher incidence of T‐cell lymphoma [3, 4, 9].

Similarly, children in subequatorial Africa are at higher risk of endemic Burkitt lymphoma due to Epstein–Barr virus (EBV), with a suspected interaction with malaria infection.

Differences in the risk of viral NHL by ethnicity and geography remain unclear [2, 15].

One of the problems with lymphomas is that the telltale symptoms are nonspecific and can be mistaken for less serious illnesses, such as the flu, which works against early diagnosis, which is so essential to improve prognosis [16, 17].

The diagnosis of lymphoma is based on the study of a tissue sample obtained from a biopsy. This involves removing a lymph node, or a fragment of a lymph node, or other suspicious tissue for histopathological, immunohistochemical, and/or biomolecular analyses [18].

Treatments for non‐Hodgkin lymphoma have been the subject of significant progress over the past two decades. Chemotherapy, radiotherapy, immunotherapy, and stem cell transplantation are used alone or in combination. Treatment strategies differ depending on whether it is an indolent or aggressive lymphoma [13].

Indolent lymphomas are very sensitive to chemotherapy, but relapses occur [19].

In Africa, estimates remain patchy, as many countries lack reliable health data management systems. However, in sub‐Saharan Africa, among hematological cancers, lymphomas are considered an important cause of morbidity and mortality [20].

In 2012 in Africa, the number of new cases of NHL was estimated at 26,224 with a mortality rate of 83% [17, 21, 22].

NHL accounted for 6.8% in men, 3.1% in women, and 20.7% in children of all cancers [1, 15, 20].

Mortality from NHL is proportionally higher in Africa than elsewhere [1, 15, 23].

In the Democratic Republic of Congo, the absence of the national cancer registry is a serious handicap to the epidemiological evaluation of NHL in the DRC, and therefore difficult to improve management because to fight well, it is necessary to know how to count. However, some studies, although fragmentary, have shown increasing frequencies of NHL cases in the DRC [24, 25, 26].

However, there is a lack of knowledge about this pathology among the population and health professionals. Requests for pathological tests are limited, and the clinical manifestations of NHL are confusing with other pathologies. An observational survey of the country's hospitals is enough to note the absence of protocols for the management of NHL.

Does the description of epidemiological aspects contribute to the improvement of the management of non‐Hodgkin's lymphomas in the DRC? Knowledge of the epidemiological aspects would contribute to the improvement of the fight against non‐Hodgkin's lymphoma in the DRC. The objective of this study is to determine the epidemiological aspects of non‐Hodgkin lymphoma in the DRC.

## 2 Materials and Methods

This is a descriptive cross‐sectional study, conducted at the University Clinics of Kinshasa (CUK), over 12 years, from January 1, 2012, to December 31, 2022.

The CUK is a tertiary‐level hospital, organized into departments (12 departments), including the Department of Pathological Anatomy. It is a health facility with 800 beds. The CUK is located in Kinshasa, in the commune of Lemba, at 15°18′51″ East longitude, 4°24′51″ South latitude, and at 441 m above sea level. Since its creation in 1957, the CUK has been a specialized framework that carries out three main missions: specialized health care, teaching, and research [27].

The study population consists of lymphoma patients whose biopsies, excision biopsies, or surgical specimens have been performed and sent to the laboratories selected for the study.

Data were collected over a period of 1 month, from July 30 to August 30, 2023, from a literature review, the CUK pathological anatomy laboratory archives, including data sheets, registers, protocols, and histopathological analysis records. As the study was on archival material and not on individuals, informed consent was not required. A linear database was created in which sociodemographic parameters (age, sex, origin of patients) and anatomo–clinical parameters (anatomical site, anatomo–clinical types, histological types, and subtypes) were included. The sex variable was represented in two modalities (male and female); the same is true for the anatomo–clinical type variable (aggressive and indolent). The other variables contained several modalities.

Statistical analyses were performed using Excel and R software. Descriptive statistics consisted of calculating frequency measures, central tendency measures, and dispersion measures.

The median with the ends (minimum and maximum) is used for skewed distributions. Qualitative variables and discrete quantitative variables are presented as proportions (%). The chi‐square correlation test is used to compare proportions with the 95% confidence interval (significance *α* = 0.05).

## 3 Results

### 3.1 Overall Results

Out of a total of 2070 cancer cases, 51 cases of non‐Hodgkin lymphoma were recorded at the CUK Pathological Anatomy Laboratory from 2012 to 2022.

55% of cases were male versus 45% female, with a sex ratio of 1.2.

The age of the patients ranged from 3 to 80 years, with a mean age of 42 years and a median age of 45 years.

57% of the cases recorded came from Kongo‐central (15 cases) and Kinshasa (14 cases).

From an anatomical and clinical point of view, 98% of the cases recorded were B‐cell lymphomas compared to 2% T‐cell lymphomas.

More than 80% of NHL cases were aggressive; 5 histological types were identified, and 11 immunophenotyping subtypes were identified.

More than half, or 59% of the cases, were lymph node involvement.

### 3.2 Description of the Sociodemographic Aspects of Patients With Non‐Hodgkin Lymphoma

The Table 1 shows the distribution of NHL cases by age and sex: Among men, the age groups ≥ 41 years were more affected, that is, 32% each, while among women, it is the ≤ 20 age group that is most affected, with 43.5% of cases.

**TABLE 1:** Distribution of NHL cases by age and sex.

Age range	Masculine		Feminine		Total	
	*n*	%	*n*	%	*n*	%
1–20	5	17.9	10	43.5	15	29.4
21–40	5	17.9	5	21.7	10	19.6
41–60	9	32.1	2	8.7	11	21.6
≥ 61	9	32.1	6	26.1	15	29.4
Total	28	100.0	23	100.0	51	100

*Note:* Among men, the age groups ≥ 41 years were more affected, that is, 32% each, while among women, it is the ≤ 20 age group that is most affected with 43.5% of cases.

**FIGURE 1:**
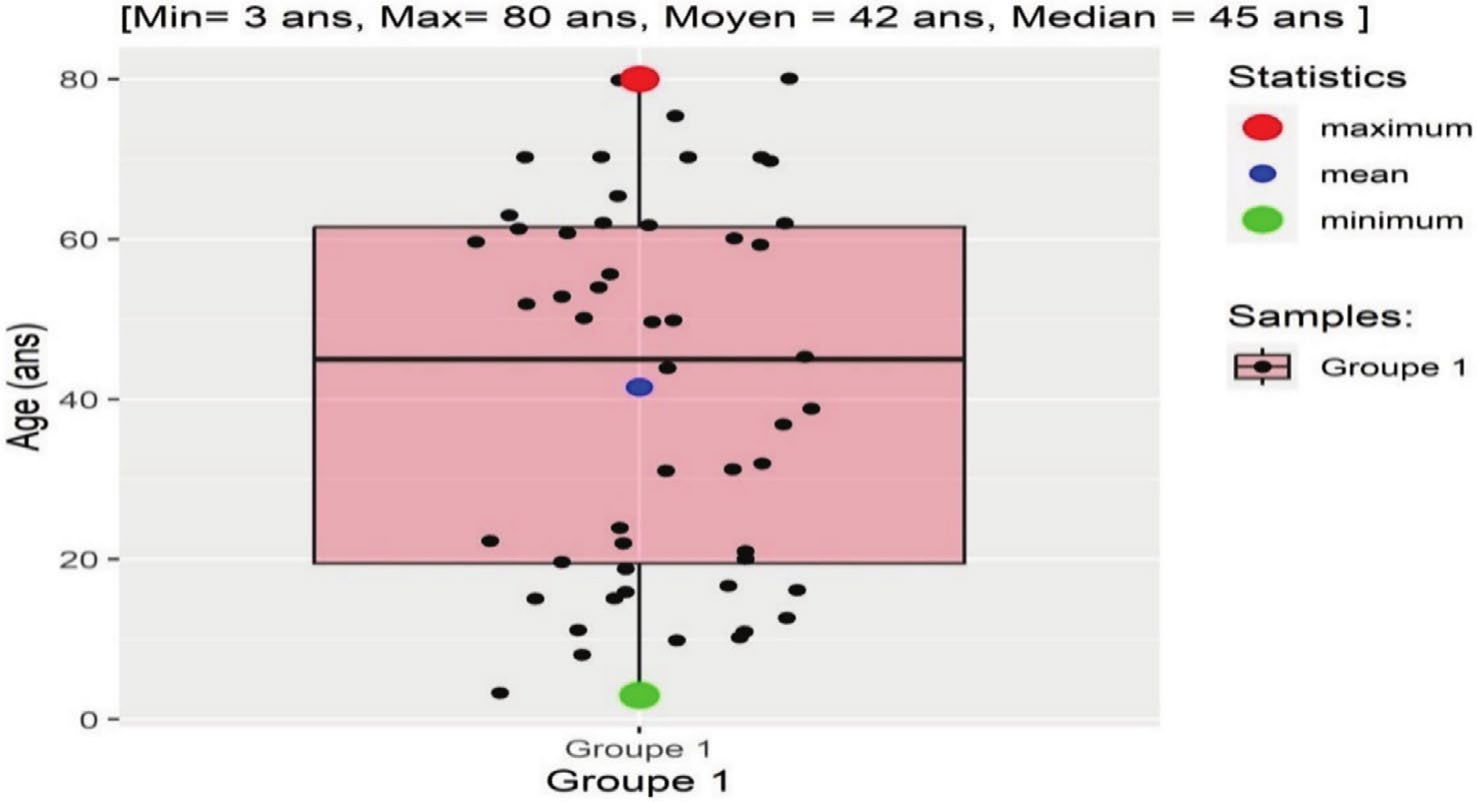
Distribution of NHL cases by age and sex. This figure shows a high distribution of cases in the extreme age groups, that is, 29% of cases for the 1–20 age group and 29% for the ≥ 61 age group.

The Figure 1 shows a high distribution of cases in the extreme age groups, that is, 29% of cases for the 1–20 age group and 29% for the ≥ 61 age group.

**FIGURE 2:**
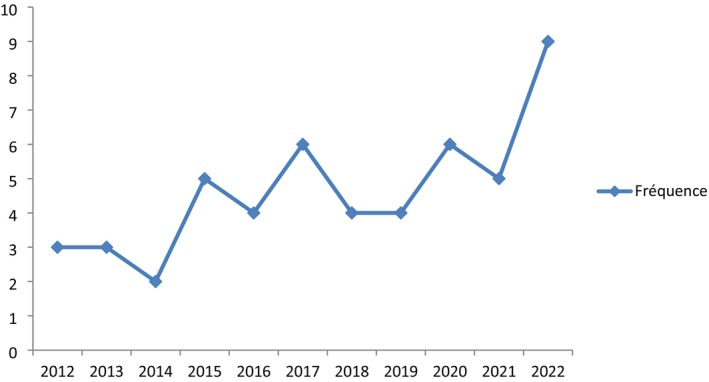
Annual change in NHL frequency. This figure shows that the year 2022 recorded more NHL cases, at 18%.

The Figure 2 *watch* annual change in NHL frequency shows that the year 2022 recorded more NHL cases, at 18%.

**FIGURE 3:**
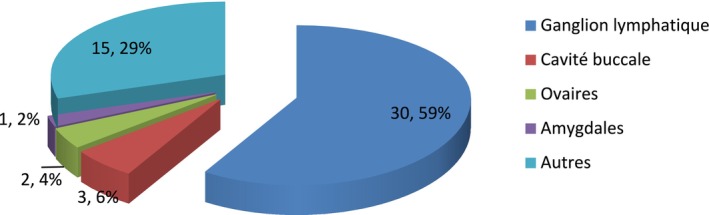
Distribution by anatomical site. The majority of NHL cases were localized to the lymph node (59%).

The Figure 3 describes distribution by anatomical site: the majority of NHL cases were localized to the lymph node (59%).

**FIGURE 4:**
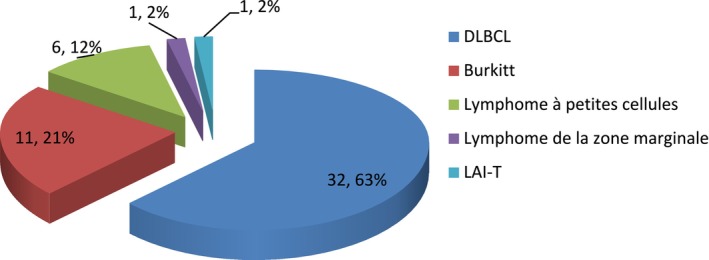
Distribution of the different histological types of NHL. The majority of NHL cases were diffuse large B‐cell lymphoma (DLBCL) (63%), followed by Burkitt's lymphoma (21%). LAI‐T (Angion‐immunoblastic T lymphoma). DLBCL diffuse large B‐cell lymphoma.

The Figure 4 describes the distribution of the different histological types of NHL: the majority of NHL cases were diffuse large B‐cell lymphoma (DLBCL) (63%), followed by Burkitt's lymphoma (21%).

Table 2 describes the distribution of NHL cases by age group by type: the DLBCL was found in all age groups with a high number of cases (12 cases) in the last age group.

**TABLE 2:** Distribution of NHL cases by age group by type.

Types LNH/Slice Age	Aggressive						Indolent			
	DLBCL		BURKITT		LAI‐T		LLC‐B		Marginal areas	
	*n*	%	*n*	%	*n*	%	*n*	%	*n*	%
1–20	6	19	8	72	0	0	1	17	0	0
21–40	6	19	3	28	1	100	0	0	0	0
41–60	8	25	0	0	0	0	3	50	0	0
≥ 61	12	37	0	0	0	0	2	33	1	100
Total	32	100	11	100	1	100	6	100	1	100

*Note:* This table shows that DLBCL was found in all age groups with a high number of cases (12 cases) in the last age group.

Abbreviations: DLBCL, diffuse large B‐cell lymphoma; LAI‐T, angion‐immunoblastic T lymphoma; LLC‐B, chronic lymphocytic leukemia.

## 4 Discussion

Out of a total of 2070 cancer cases, 51 cases of non‐Hodgkin lymphoma were recorded at the CUK Pathological Anatomy Laboratory from 2012 to 2022, which represents a frequency of 2.5% of NHL cases compared to all cancers. Although fragmented, this frequency is underestimated, on the one hand by the high cost of immunohistochemical analyses, which limits access to diagnosis for the poor population, and on the other hand by the lack of knowledge among the population about this disease, the signs of which are confusing with other diseases. These results confirm data from the literature that report nearly 3% of the frequency of NHL over the entire cancer [1, 7, 11, 16].

The increase in NHL frequency observed at the study period would also be due to improved detection and diagnosis tools, which justifies the progression curve from 2012 to 2022.

The proportion of NHL to all lymphomas in this study was 78%. This proportion has been reported in the literature and the work of several authors to about 80% of NHL cases in all lymphomas [1, 2, 7, 20].

About 98% of the recorded cases were B‐cell lymphomas compared to 2% of T‐cell lymphomas. The infectious factors implicated in B NHL are the most common in the DRC, which would justify this high frequency. These results support data from the literature that report a high frequency of more than 80% of B‐cell lymphomas [1, 16].

The proportion of type B NHL is higher in sub‐Saharan Africa than in the West [5, 19, 24].

The high incidence of type B NHL in some parts of Africa is linked to infectious causes, including viral and parasitic causes [20, 24].

55% were male compared to 45% female, with an M/F sex ratio of 1.2. These results are similar to those in the literature that report a predominance of NHL cases in males, with a sex ratio around 1 [1, 16, 19, 24].

The age of the patients ranged from 3 to 80 years, with the mean age of 42 years and the median age of 45 years. The extreme age groups were equally affected, with 29% for the 1–20 age group and 29% for the over 60 age group. The high frequency of NHL cases in the age group of 1–20 years is linked to the Congolese population, which is mostly young, and to the number of high cases of Burkitt's lymphoma (endemic type) as well as the outbreak of infectious and environmental agents, which are ubiquitous and closely linked to hygiene conditions in this age group.

These findings support those in the literature that report that almost all NHLs in children are aggressive [1, 2, 7].

The incidence changes with age; other authors have reported a peak in the range 0–40 years and in the 11–20 years range [6, 24, 25].

In general, the average age of NHL in developing countries is lower than in Western countries, which is between 50 and 60 years [1, 7, 19].

The results obtained reported a predominantly lymph node location, that is, 59% of cases. These results support those in the literature that report lymph node predominance [11, 16, 19, 20].

86% of NHL cases recorded were of the aggressive type. The infectious and environmental factors implicated in aggressive‐type NHL would justify the high frequency found in the present study. These results confirm those in the literature that report a predominance of aggressive NHL [7, 18, 24].

Numerous studies in Africa have shown a very high frequency of aggressive lymphomas, particularly in Côte d'Ivoire, with 81% [14, 15, 28, 29].

In addition, these are almost always B‐cell lymphomas and more rarely T‐cell lymphomas, which are more aggressive [15, 30].

The frequencies found are superimposed on those of the authors who also report a predominance of aggressive lymphomas [25, 26, 30].

The results of the present study reported a predominance of diffuse large B‐cell lymphomas (DLBCL) at 63% with the predominant NOS subtype at 27%, followed by the immunoblastic subtype (23%).

The limited use of immunostaining techniques, on the one hand due to a lack of resources and on the other hand due to the lack of certain markers, sometimes makes it difficult to make an accurate diagnosis, with the subtyping of NHL in the DRC. These results are higher than data from some authors who found a predominance of diffuse large B‐cell lymphoma in 32%–34% of cases [16, 19, 24].

The late diagnosis would be the cause of the high proportions of DLBCL observed, since other indolent forms are often asymptomatic and evolve toward the symptomatic aggressive forms, including DLBCL, which motivates the patient to consult.

The frequencies found are superimposed on those of other authors who also report a predominance of aggressive lymphomas, including 74.6%, 82.5%, and 83% [25, 26, 30].

The results of the present study showed that, at a 95% confidence interval, the anatomical clinical type is statistically dependent on age, with a *p* value of 2.2 × 10^−16^. This could be explained by the high frequency of cases of aggressive NHLs in certain groups. These results are superimposed on those in the literature that report more cases of aggressive types of NHL cases in children [2, 7, 31].

## 5 Conclusion

Non‐Hodgkin lymphoma (NHL) is a heterogeneous group of cancers whose global incidence has been increasing in recent years, with the outbreak of environmental and infectious factors.

The clinical manifestations of this group of diseases are often confused with other pathologies, which work against early diagnosis, which is so essential to improve prognosis. Only histopathological, immunohistochemical, and/or biomolecular analyses can be used to make the diagnosis with typing and subtyping before the initiation of treatment.

In the Democratic Republic of Congo, there is still a lack of knowledge among the population and health professionals about this disease.

The absence of the National Cancer Registry is a serious handicap to the epidemiological evaluation of NHL.

The socioeconomic impasse of the population, the difficulty of access to health care, remains a bottleneck for people suffering from NHL, who often seek consultation at the late stage of the disease.

The high cost of these tests, combined with the precarious socioeconomic conditions of the population, means that many people with signs related to this type of disease do not have access to appropriate care.

Knowledge of epidemiological aspects contributes to improving the fight against non‐Hodgkin lymphoma in the DRC.

This study has some limitations due to its literature review nature; some patient clinical information was not provided, and the records do not always indicate the diagnosis at discharge. A much larger, multicenter study, integrating several ecological, sociodemographic, and anatomical–clinical parameters, will provide a clear idea of the epidemiological profile of NHL in the DRC.

The combined efforts of all the actors involved in the fight against NHL, with the involvement of the CNLC for the provision of a national cancer registry, the connectivity between different structures specialized in the treatment of NHL, the implementation of a protocol for the management of NHL, as well as the subsidy of histopathological and immunohistochemical analyses.

## Author Contributions

**Mbwamulungu Nakweti Julia:** conceptualization, methodology, data curation, software, formal analysis, funding acquisition, resources, writing – original draft, writing – review and editing. **Azako David:** formal analysis, project administration. **Pezo Serge:** data curation. **Bokambadja Fabrice:** validation, data curation. **Kapour Kieng Germain:** methodology, validation, investigation, supervision. **Bompangue Nkoko Didier:** validation, supervision, project administration, resources. **Kisile Olive:** validation. **Lebwaze Massamba Bienvenu:** conceptualization, methodology, supervision, validation, project administration. **Kabongo Mpolesha Jean‐Marie:** conceptualization, methodology, validation, supervision, project administration, resources.

## Conflicts of Interest

The authors declare no conflicts of interest.

## Data Availability

The data that support the findings of this study are available on request from the corresponding author. The data are not publicly available due to privacy or ethical restrictions.
